# Tanshinone IIA Inhibits Triple-Negative Breast Cancer Cells MDA-MB-231 via G Protein-Coupled Estrogen Receptor- (GPER-) Dependent Signaling Pathway

**DOI:** 10.1155/2023/8371623

**Published:** 2023-01-27

**Authors:** Yueshuang He, Ke Yang, Jiao Liu, Danning Shi, Zeye Zhang, Jiadi Yang, Meng Chen, Piwen Zhao

**Affiliations:** ^1^School of Life Sciences, Beijing University of Chinese Medicine, No. 11 East Road, North 3rd Ring Road, Beijing 100029, China; ^2^School of Chinese Medicine, Beijing University of Chinese Medicine, No. 11 East Road, North 3rd Ring Road, Beijing 100029, China

## Abstract

Due to the lack of classic estrogen receptors, there has been a shortage of targeted therapy for triple-negative breast cancer (TNBC), resulting in a poor prognosis. However, the newly discovered G protein-coupled estrogen receptor (GPER) has been found to be expressed in TNBC cells. *Salvia miltiorrhiza* (Danshen) is an essential Chinese medicine for gynecological disorders, and its component tanshinone IIA (Tan IIA) exerts an anticancer effect. Therefore, this study attempted to investigate whether GPER is involved in the inhibitory effect of Tan IIA on TNBC. We applied various databases and GO pathway analysis to predict the possible mechanism of Tan IIA. We identified 39 overlapping targets, including c-Jun, c-Fos, and caspase-3, and enriched cell cycle-related pathways. Next, we demonstrated the strong binding ability of Tan IIA to GPER by molecular docking assay. In the subsequent validation tests, Cell Counting Kit-8 (CCK8) assay showed that Tan IIA inhibited proliferation of MDA-MB-231 cells time and dose dependently without affecting normal cells. Using Transwell plate, flow cytometry, and Western blot assays, we showed that Tan IIA inhibited migration and induced apoptosis of MDA-MB-231 dose dependently. Importantly, protein expressions of GPER, epidermal growth factor receptor (EGFR), extracellular regulated protein kinases (ERK), c-Fos, and c-Jun were all decreased by Tan IIA dose dependently. Administration of GPER inhibitor partly abolished these effects. Furthermore, nuclear translocation of c-Fos and c-Jun as well as cell cycle-related proteins was downregulated by Tan IIA dose dependently. In summary, Tan IIA could inhibit the proliferation and migration of MDA-MB-231 cells and induce apoptosis, and the possible mechanism may be the regulation of GPER-mediated pathways, suggesting that GPER could be a therapeutic target for TNBC.

## 1. Introduction

Triple-negative breast cancer typically occurs in premenopausal women aged 40 to 55 years old and is defined by the absence of receptor estrogen (ER), progesterone (PR), and human epidermal growth factor receptor-2 (HER2) [1]. It is highly aggressive and resistant to chemotherapeutic agents. Approximately 46% of TNBC patients develop distant metastases with a 5-year survival rate less than 30% [2].

It is intractable to treat TNBC due to the lack of a specific targeted therapeutic regimen. GPER is a recently identified novel estrogen receptor. It is expressed in TNBC cells and is closely associated with the development of breast cancer [3]. It was reported that GPER plays a critical role in tumorigenesis through Hippo/YAP/TAZ pathway [4]. In addition, GPER and downstream PKA/BAD signaling maintain the stemness of breast cancer stem cells [5]. GPER could mediate the activation of epidermal growth factor receptor (EGFR), mitogen-activated protein kinase (MAPK), and phosphatidylinositol 3-kinase/protein kinase B (PI3K/AKT) [6]. Among them, EGFR is thought to be correlated with multiple types of tumors, and it is upregulated in TNBC with an indication of poor prognosis [7]. In breast cancer cells, GPER agonists were shown to increase survival of tumor cells through activating EGFR/ERK pathway [8].

Traditional Chinese medicine (TCM) has a long history of application in the prevention and treatment of complex diseases [9], and in clinical practice, *Salvia miltiorrhiza* could be used in breast cancer therapy [10]. It contains a number of diterpene quinone and phenolic acid derivatives, such as Tanshinone, cryptotanshinone, isocryptotanshinone, miltirone, tanshinol, and salviol [11]. Among them, Tan IIA was reported to exhibit a remarkable antibreast cancer effect [12–15]. Our previous study demonstrated that GPER-mediated PI3K/AKT signaling pathways are involved in the inhibitory effect of cryptotanshinone on the proliferation of breast cancer SKBR-3 and MCF-7 [16, 17]. In this context, it can be questioned whether GPER is also involved in the antitumor property of Tan IIA.

Network pharmacology is a drug research tool and has been extensively employed in TCM to uncover the molecular mechanisms underlying their efficacy [18]. In the current study, we applied this method to predict the possible mechanisms of Tan IIA against breast cancer cells, and *in vitro* tests were subsequently performed for validation.

## 2. Methods

### 2.1. Targets of Tan IIA

The structured data format of Tan IIA was obtained from PubChem database (https://pubchem.ncbi.nlm.nih.gov/) [19] and then was imported into SwissTargetPrediction database (http://www.swisstargetprediction.ch/) [20]. The outputs with prediction scores greater than 0 were taken as potential targets. Meanwhile, the targets of Tan IIA were also retrieved from TCMSP (https://old.tcmsp-e.com/tcmsp.php) [21], and target names were standardized using UniProt database (http://www.uniprot.org/) [22].

### 2.2. Targets of TNBC

Targets of TNBC were retrieved from the following resources with the keyword “Triple negative breast cancer”: DrugBank database (http://www.drugbank.ca/) [23], GeneCards (http://www.genecards.org/) [24], and Therapeutic Target Database (http://bidd.nus.edu.sg/group/cjttd/) [25]. TNBC targets were compared with the predicted Tan IIA targets, and Venny 2.1.0 (https://bioinfogp.cnb.csic.es/tools/venny/index.html) [26] was used to draw the common targets.

### 2.3. Protein-Protein Interaction

Common targets were imported into Cytoscape software to map the “component-target-disease” interaction network. On the other hand, common targets were also imported into STRING (https://string-db.org/) [27] to construct a protein-protein interaction (PPI) network model, with the species limited to “Homo sapiens” and a confidence score > 0.4 [28].

### 2.4. Pathway and Functional Enrichment Analyses

Key target gene GO functional enrichment analysis was performed based on R software using Bioconductor bioinformatics package with *P* value < 0.05 and *Q* value < 0.05. Results were output as bar graphs and bubble plots.

### 2.5. Materials

Tan IIA (purity > 98% and molecular weight 294.34) was purchased from National Institutes for Food and Drug Control (Beijing, China). 2.943 mg Tan IIA was dissolved in 1 mL dimethyl sulfoxide (DMSO, Sigma, USA) to make a 1 × 10^4^ *μ*M stock solution, which was then added to the medium at the indicated concentrations. Cell Counting Kit-8 (CCK-8) was purchased from Abcam (USA). Fetal bovine serum and 0.05% trypsin were from Gibco (USA), and L15 medium was from HyClone (USA). GPER specific antagonist G-15 was from Cayman Chemical (Michigan, USA). The following antibodies were used: GPER, EGFR, ERK1/2, cyclin A2, cyclin D1, CDK2,4,6, caspase-3, lamin B1 (Abcam, USA), c-Fos, and c-Jun (Proteintech Group, USA).

### 2.6. Cell Culture

Human breast cancer cell line MDA-MB-231 (National Infrastructure of Cell Line Resource, Beijing, China) was cultured in L15 glucose medium supplemented with 10% fetal bovine serum at 37°C in a non-CO_2_ atmosphere. HEK293 cells (National Infrastructure of Cell Line Resource, Beijing, China) were cultured in DMEM glucose medium supplemented with 10% fetal bovine serum at 37°C in CO_2_ atmosphere. 100 *μ*g/mL streptomycin and 100 U/mL penicillin were added to avoid contamination.

### 2.7. Cell Proliferation Assay

Cells were seeded at the density of 2 × 10^4^ cells per well in 96-well plates and grew overnight. MDA-MB-231 cells were treated with Tan IIA at the concentrations of 0, 1, 5, 10, 50, or 100 *μ*M, respectively, for 24 h, 48 h, and 72 h. HEK293 cells were treated with Tan IIA at the concentration of 5, 10, or 50 *μ*M for 48 h. 0.2% DMSO was used as a control. After drug administration, 100 *μ*L CCK8 (1 mg/mL) was added and incubated for 2 h in the dark. Subsequently, absorbance was measured at 450 nm using a multifunctional enzyme marker (Multiskan GO, Thermo Fisher Scientific, USA).

### 2.8. Migration Assays

Cell migration ability was assayed by Transwell plates (Corning, NY, USA). Cells at logarithmic growth phase were centrifuged and resuspended in medium containing 5% FBS, and the cell density was adjusted to 1 × 10^5^ cells/mL. 100 *μ*L of cell suspension was inoculated in the upper chamber of the Transwell, and 750 *μ*L of medium containing 10% FBS was added to the lower chamber. After attached, the cells were incubated with Tan IIA at concentrations of 0, 5, 10, G15+10, or 50 *μ*M in each well. The cells were incubated for 48 h, then the upper chamber was aspirated, and 4% paraformaldehyde (Solarbio, Beijing, China) was added and fixed at room temperature for 20 min. 0.1% crystal violet staining solution (Solarbio, Beijing, China) was added and stained at room temperature for 20 min. The upper chamber was gently rinsed with running water and soaked three times for decolorization and staining, the upper chamber was aspirated, and the residual water in the upper chamber was gently wiped off with a cotton swab. The experiment was repeated three times.

### 2.9. Apoptosis Rate Analysis

Cells were seeded overnight in 6-well plates at a density of 3 × 100000 per well for adhesion. Changed the medium with 0, 5, 10, G15+10, or 50 *μ*M Tan IIA and incubated for 48 h. Collect cells and added 500 *μ*L binding buffer to get single-cell suspension. Each group was stained with 5 *μ*L Annexin V-FITC for 10 min and then 5 *μ*L PI for 5 min avoiding light at room temperature. Within 1 h, apoptosis rate was determined by flow cytometry for three times.

### 2.10. Western Blot Assay

Total protein was obtained from treated cells in RIPA buffer (Applygen, Beijing, China) supplemented with 1% protein phosphatase inhibitor (all-in-one, 100x, Solarbio, Beijing, China). Proteins were centrifuged at 12,000 rpm and 4°C for 5 min. The protein concentration is 30 *μ*g/*μ*L. Separate via sodium dodecyl-sulfate polyacrylamide gel electrophoresis and transfer onto polyvinylidene fluoride membranes (Millipore, Bedford, MA, USA). Block polyvinylidene fluoride membranes in 5% bovine serum albumin (Gen-view Scientific Inc., USA) for 1.5 h at room temperature. Western blot analyses were performed using the following primary antibody dilutions: rabbit anti-human GPER (1 : 250), EGFR (1 : 5000), ERK1/2 (1 : 1000), cyclin A2 (1 : 1000), cyclin D1 (1 : 10000), CDK2 (1 : 5000), CDK4 (1 : 2000), CDK6 (1 : 50000), caspase-3 (1 : 10000), c-Jun (1 : 3000), lamin B1 (1 : 5000), mouse anti-c-Fos (1 : 1000), and GAPDH (1 : 5000). Next, the membranes were incubated with horseradish peroxidase-conjugated secondary antibodies (Proteintech, USA) at room temperature for 1 h. Subsequently, the Enhanced Chemiluminescence Detection Kit was used to detect and visualize protein bands.

### 2.11. Molecular Docking

In order to further verify the interaction between active components of Tan IIA and GPER, computer molecular docking technology was carried out. The conformation of GPER was retrieved from the UniProt database. The resulting conformation was a seven-helix structure. Subsequently, the crystal structure of GPER obtained from GPCR-I-TASSER was optimized by PyMOL and AutoDock tool software for the later docking. The structure of Tan IIA is built by PubChem on its chemical structure, respectively. A flexible docking was carried out by AutoDock Vina, and the grid box was covered on the protein to make a blind docking. Visualization of docking results was built using PyMOL and Discovery Studio.

### 2.12. Statistical Analysis

Data was analyzed using SPSS 20.0. Results of three independent tests were presented as mean ± SD. One-way ANOVA followed by the LSD-*t* multiple comparison test was performed among multiple groups. Unless stated otherwise, *P* values < 0.05 were considered significant.

## 3. Results

### 3.1. Network Pharmacology Predicted the Possible Mechanisms of Tan IIA on TNBC

**Figure 1:**
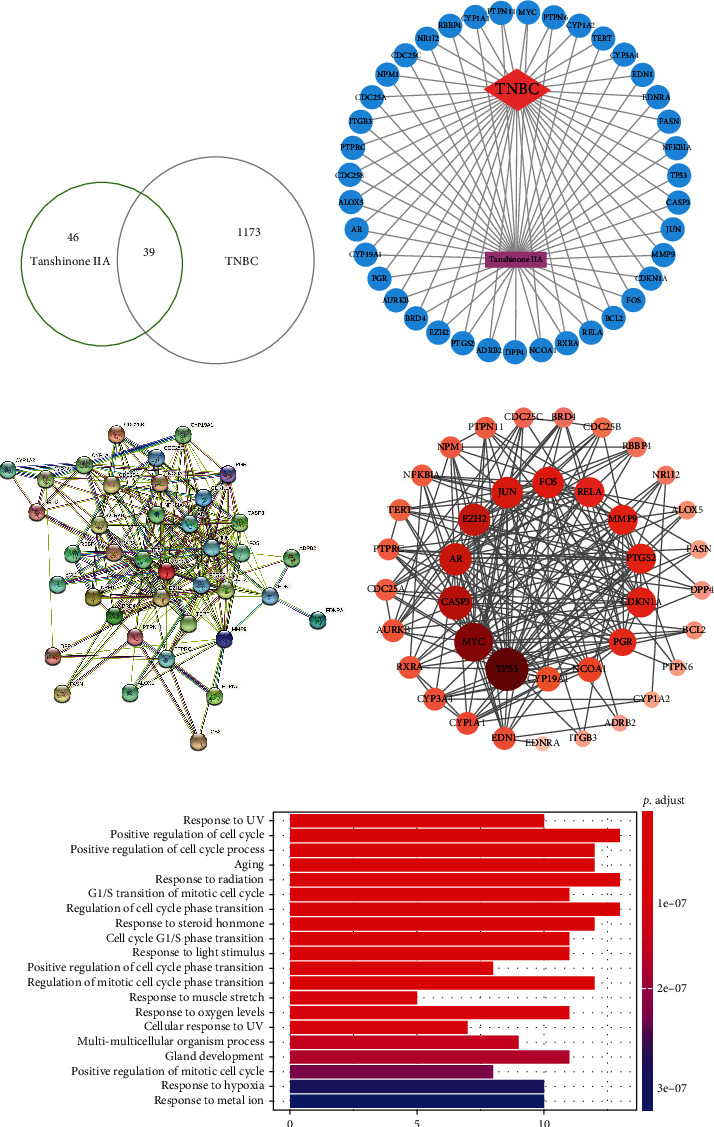
Network construction. (a) Common targets screened from Tan IIA and TNBC. (b) Tan IIA-target-TNBC network. Blue circles represent potential targets of Tan IIA for the treatment of TNBC. (c, d) Protein-protein interaction (PPI) network constructed by STRING database and Cytoscape software. In Figure 2(d), the size of the node, its color, and its shade variation represent the magnitude of the degree value. (e) GO enrichment analysis of 39 common targets. *P* adjust represents the significance of enrichment. The redder the color, the higher the enrichment.

85 drug targets were retrieved from both SwissTargetPrediction and TCMSP databases, and 1212 TNBC genes were obtained. Overlapping 39 targets were identified (Figure 1(a)) and further imported into Cytoscape software for “component-target-disease” interaction network construction. Purple color represents Tan IIA, blue color represents 39 common targets, and red color represents the diseases (Figure 1(b)). Protein-protein interactions were also identified by STRING database, where the size, color, and shade of the nodes represent the degree value. Caspase-3, c-Fos, and c-Jun were selected for further in vitro experiments (Figures 1(c) and 1(d)). 39 common targets were run through R software, and then, GO analysis was performed. The mainly enriched pathways include cell cycle-related pathways (Figure 1(e)).

### 3.2. Tan IIA Inhibited MDA-MB-231 Cell Growth

**Figure 2:**
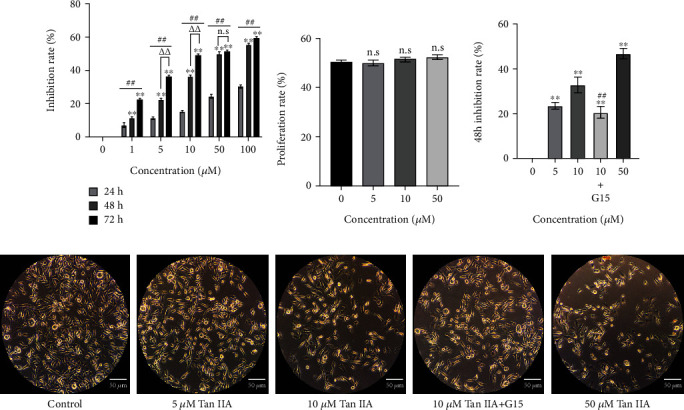
The effects of Tan IIA on cell proliferation. (a) MDA-MB-231 cells were treated with different concentrations of Tan IIA for 24 h, 48 h, or 72 h, and cell proliferation was tested using CCK8 assay kits; cell inhibition rate = 1- cell proliferation rate. ^∗∗^ represents *P* values < 0.01 compared to 24 h of intervention, ## represents *P* values < 0.01 compared to 0 *μ*M Tan IIA, △△ represents *P* values < 0.01 compared to 48 hours of intervention, and n.s represents no statistical difference compared to 48 hours of intervention. (b) Tan IIA did not affect proliferation of normal cells. (c) Addition of the specific GPER inhibitor G15 attenuated the inhibitory effect of Tan IIA on MDA-MB-231 cells. ^∗∗^ represents *P* values < 0.01 compared to control, and ## represents *P* values <0.01 compared to 10 *μ*M Tan IIA. (d) MDA-MB-231 cell morphology under inverted microscope (magnification, ×200). Scale bar represents 100 *μ*m. All results were presented as means of three independent tests ± SD. Each independent experiment was repeated six times.

We tested five concentrations and three time points of intervention and found that Tan IIA inhibited breast cancer cells in a dose- and time-dependent manner (Figure 2(a)). Considering the effectiveness of inhibition as well as cell morphology, we chose 5, 10, and 50 *μ*M concentrations of Tan IIA as the low, medium, and high doses and 48 h as the intervention duration for subsequent experiments. To test the toxicity of Tan IIA, we administrated 5, 10, or 50 *μ*M of Tan IIA to HEK293 cells for 48 h. The results showed no significant difference in the proliferation rate (Figure 2(b)), indicating that selected doses have no obvious toxicity. Next, to demonstrate that the inhibitory effect of Tan IIA is mediated by GPER, we introduced GPER specific inhibitor G15. The inhibitory capacity of Tan IIA was attenuated after addition of G15 in the middle dose group (Figure 2(c)).  Figure 2(d) is the representative images of cells.

### 3.3. Tan IIA Induced Cell Apoptosis of MDA-MB-231

**Figure 3:**
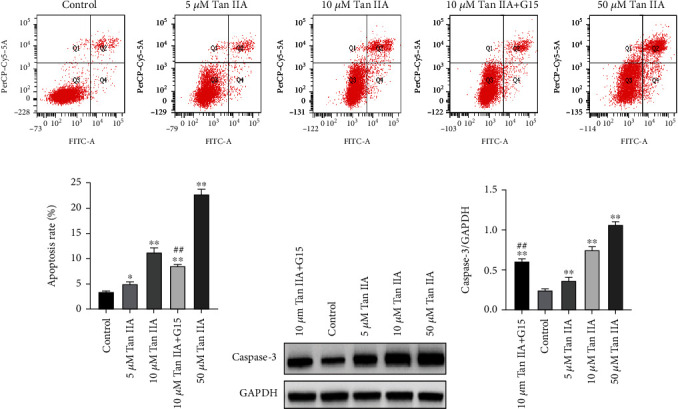
The effects of Tan IIA on cell apoptosis. (a, b) The apoptosis rate of MDA-MB-231 cells treated with 0, 5, 10, G15+10, and 50 *μΜ* Tan IIA for 48 h was detected by flow cytometry after staining with Annexin V-FITC/PI. *X* coordinate represents Annexin-FITC signal, and *Y* coordinate represents PI signal. The third quadrant is normal cells, the fourth quadrant is early apoptotic cells, the first quadrant is intermediate to late apoptotic, and the second quadrant is necrotic cells. (c, d) Effect of Tan IIA on the expression of apoptosis protein caspase-3 in MDA-MB-231 cells. ^∗∗^*P* < 0.01 compared with control, ^##^*P* < 0.01 compared with 10 *μ*M Tan IIA. All results were presented as means of three independent tests ± SD. Each independent experiment was repeated three times.

We next examined the effect of the Tan IIA on apoptosis and also added G15 to investigate the involvement of GPER. Results showed that apoptosis was induced by all three doses of Tan IIA after 48 hours of administration, and G15 significantly reduced apoptosis rate compared with 10 *μ*M Tan IIA alone. Quantitatively, the apoptosis rate was 3.43 ± 0.12%, 5.03 ± 0.33%, 11.3 ± 0.70%, 8.63 ± 0.20%, and 22.83 ± 0.78% with the concentration of 0, 5, 10, G15+10, or 50 *μ*M, respectively (Figures 3(a) and 3(b)).

Caspase-3, a marker for apoptosis, is one of the targets screened by network pharmacology [29]. Consistently, Western blot showed that Tan IIA upregulated expression of caspase-3 compared with the control group in a dose-dependent manner, and caspase-3 was decreased after addition of G15 (Figures 3(c) and 3(d)). These results suggested that the effect of tanshinone IIA on apoptosis may be mediated by GPER.

### 3.4. Tan IIA Inhibited Migration of MDA-MB-231

**Figure 4:**
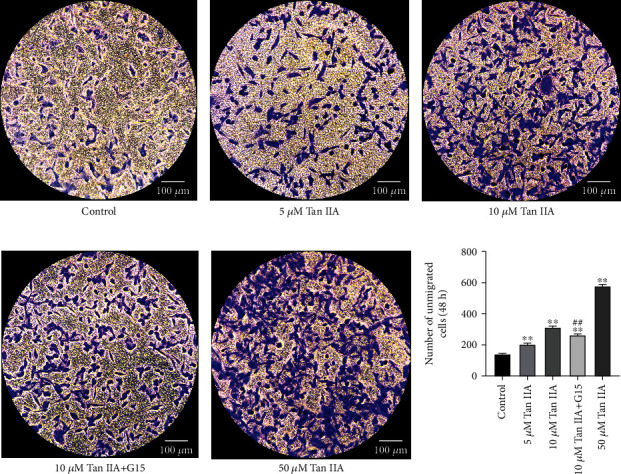
The effect of Tan IIA on the migration. (a–e) Cell migration was analyzed by Transwell. MDA-MB-231 cells were treated with 0, 5, 10, G15+10, or 50 *μ*M Tan IIA for 48 h. Representative images of nonimmigrated cells in the upper chamber were examined by crystalline violet staining. The original magnification was ×200. (f) Cells that did not migrate were counted. Data represent the mean ± SD of three independent experiments, each of which was repeated three times. ^∗∗^*P* < 0.01 compared with control and ^##^*P* < 0.01 compared with 10 *μ*M Tan IIA.

We used Transwell assay to assess the effect of Tan IIA on the migration. Cells were treated with 0, 5, 10, G15+10, or 50 *μ*M of Tan IIA and then were allowed to migrate in the Transwell chamber for 48 hours. Tan IIA increased the number of nonimmigrated cells in a dose-dependent manner compared to the control group (*P* < 0.01), while the number of nonimmigrated cells in the G15+10 *μ*M Tan IIA group was significantly lower compared to that in the 10 *μ*M Tan IIA group (*P* < 0.01) (Figure 4). These results suggested that inhibited migration of MDA-MB-231 cells by Tan IIA may be mediated by GPER.

### 3.5. GPER/EGFR/ERK/c-Fos and c-Jun Signaling Were Involved in the Activity of Tan IIA

**Figure 5:**
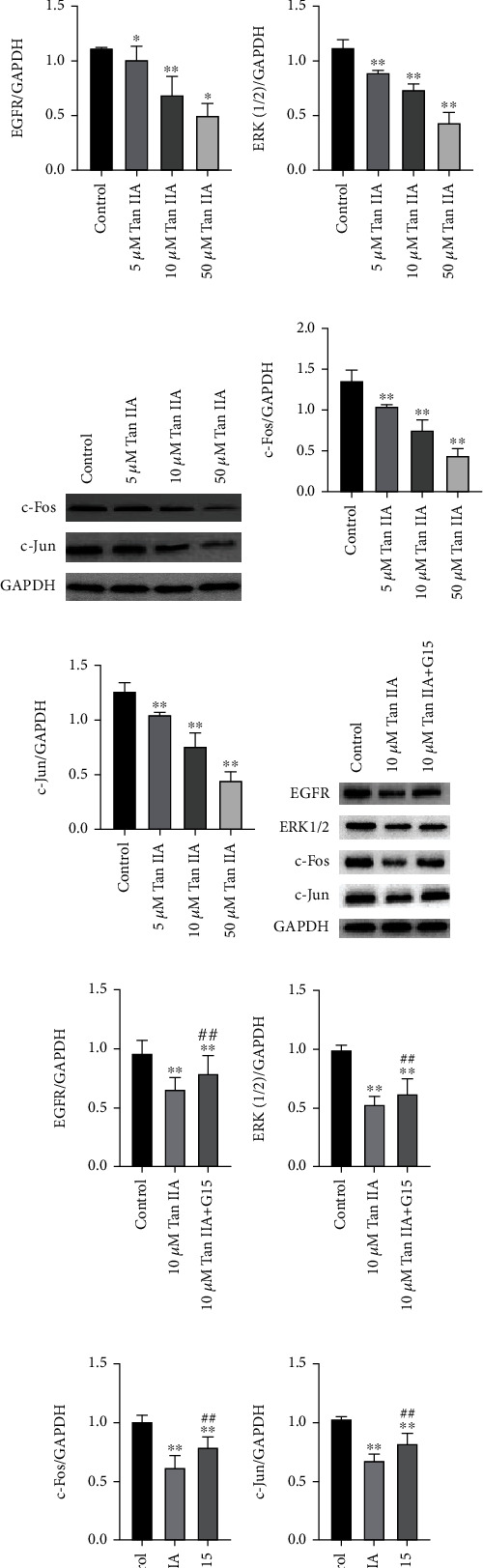
The effect of Tan IIA on EGFR/ERK (1/2) signaling pathway. (a–h) The expression of GPER, EGFR, ERK1/2, c-Fos, and c-Jun following treatment with Tan IIA. (i–l) The expression of EGFR, ERK1/2, c-Fos, and c-Jun following G-15 treating together with 10 *μ*M Tan IIA. The results were means of three independent replicates ± SD. Each experiment was repeated three times. ^∗∗^*P* < 0.01 or ^∗^*P* < 0.05 was considered as statistically significant compared with the control group.

To further investigate the mechanism, we examined the expression of GPER-related proteins. Western blot results showed that compared with the control group, GPER, EGFR, ERK1/2, c-Fos, and c-Jun were all downregulated by Tan IIA dose dependently (Figures 5(a)–5(g)). After adding G15 in 10 *μ*M Tan IIA, the expression of these proteins was lower compared with the control group but higher compared with 10 *μ*M Tan IIA alone (*P* < 0.01) (Figures 5(h)–5(l)). These results further confirmed that activities of Tan IIA may be dependent on GPER-mediated EGFR/ERK/c-Fos/c-Jun signaling pathway.

### 3.6. Tan IIA Inhibited Nuclear Translocation of c-Fos and c-Jun

**Figure 6:**
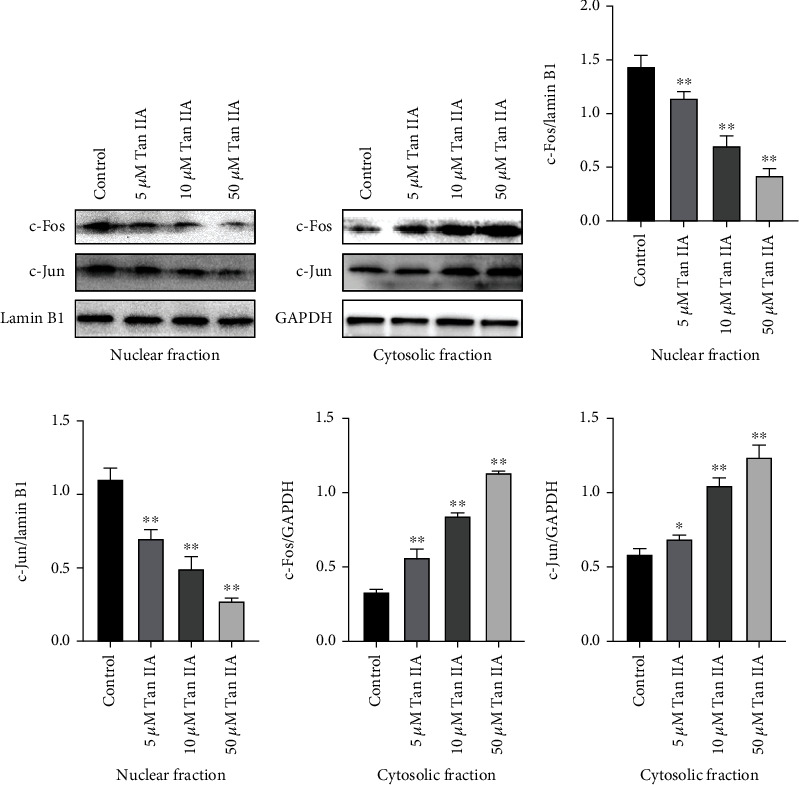
The effects of Tan IIA on c-Jun and c-Fos nuclear translocation. Nuclear and cytosolic proteins were extracted in MDA-MB-231 cells to explore the nuclear transport changes of c-Fos and c-Jun through Western blot technique after, respectively, treated with 0, 5, 10, or 50 *μ*M Tan IIA for 48 h. (a, b) Representative images of Western blotting for c-Fos and c-Jun in the nuclear and cytosolic extracts, respectively. (c–f) The quantification of the two proteins. Data were expressed as mean ± SD of three independent experiments. ^∗^*P* < 0.05 and ^∗∗^*P* < 0.01 compared with the control.

Previous study has shown that nuclear translocation of c-Fos and c-Jun affects cell migration [30]. As shown in Figure 6, in MDA-MB-231 cells, the levels of c-Jun and c-Fos proteins in the nucleus were significantly reduced by Tan IIA treatment (Figures 6(a), 6(c), and 6(d)), whereas increased levels of c-Jun and c-Fos proteins were found in cytoplasmic extracts of Tan IIA-treated cells (Figures 6(b), 6(e), and 6(f)).

### 3.7. Tan IIA Regulated the Expression of Cell Cycle-Associated Proteins

**Figure 7:**
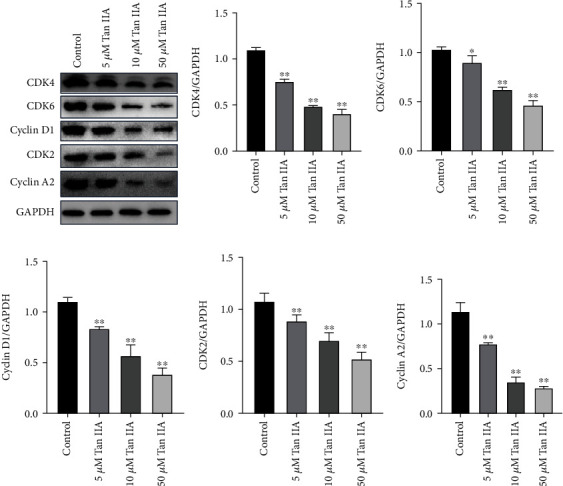
Effects of Tan IIA on the activity and expression levels of cell cycle-associated proteins. (a) MDA-MB-231 cells were treated with 0, 5, 10, or 50 *μ*M Tan IIA for 48 h, respectively. Next, Western blot was used to detect changes in the expression levels of cell cycle-associated key proteins. The band images of cyclin A2, cyclin D1, CDK2, CDK4, CDK6, and GAPDH proteins were captured via a special exposure machine. (b–f) Statistical analysis results of protein expression levels of cyclin A2, cyclin D1, CDK2, CDK4, and CDK6. Charts revealed that the protein expression of cyclin A2, cyclin D1, CDK2, CDK4, and CDK6 decreased significantly with the increase of Tan IIA concentration. Data were expressed as mean ± SD of three independent experiments. ^∗^*P* < 0.05 and ^∗∗^*P* < 0.01 compared with control.

Based on network pharmacology, we tested the expression of proteins associated with G1 and S phases by Western blot. The results showed that following Tan IIA treatment for 48 h, the expressions of cyclin A2, cyclin D1, CDK2, CDK4, and CDK6 were all decreased in a dose-dependent manner (Figure 7).

### 3.8. Tan IIA Could Bind to GPER

**Figure 8:**
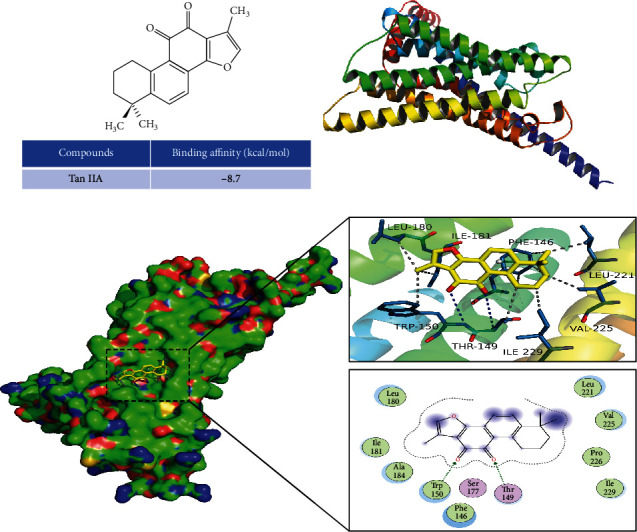
Molecular docking. (a) Chemical structure of Tan IIA. (b) Structure of GPER. (c) Docking pattern of Tan IIA with GPER. The protein structure is shown in green, blue, and yellow (ribbon); Tan IIA is shown in yellow carbon. The blue lines represent the hydrogen bond between Tan IIA and GPER; the grey line represents the hydrophobic bond between Tan IIA and GPER.

Molecular docking showed that the affinity of Tan IIA-GPER was -8.7 kcal/mol, which indicates that Tan IIA could strongly bind to GPER (Figure 8(a)). Visualization results showed that Tan IIA could bind to amino acid residues PHE146, THR149, TRP150, LEU180, ILE181, LEU221, VAL225, and ILE229 on GPER as hydrophobic bonds and to THR149 and TRP150 on GPER as conventional hydrogen bonds (Figures 8(b) and 8(c)).

## 4. Discussion

In this study, we first used network pharmacology method to preliminary explore the possible mechanism of action of Tan IIA on breast cancer and identified c-Fos and c-Jun as the potential targets. Since our previous study has shown that cryptotanshinone could inhibit MCF-7 and SKBR-3 cells through GPER pathway and c-Fos as well as c-Jun are downstream of GPER, we intend to investigate whether GPER is also involved in the efficacy of Tan IIA on another breast cancer cell line MDA-MB-231. Through molecular docking assay, we found that Tan IIA could bind closely to GPER. Through CCK8, Western blot, flow cytometry, and Transwell tests, we demonstrated that Tan IIA could inhibit the proliferation and migration of MDA-MB-231 cells and induce their apoptosis, and GPER/EGFR/ERK signaling pathway may be involved.

One of the main benefits of Chinese medicine is that it has relatively fewer toxic side effects [31–33]. In this study, Tan IIA successfully inhibited tumor cells and did not affect the proliferation rate of normal cell HEK293, confirming the advantages of Chinese medicine at the cellular level. The physiological homeostasis between cell apoptosis and proliferation is critical for tumor growth, and here, we demonstrated that Tan IIA could inhibit tumor cells from both sides. Besides, caspase-3, a critical biomarker for apoptosis, was predicted as a target, and the following in vitro test verified that Tan IIA could increase its expression. The important reasons for the poor prognosis of TNBC are its highly aggressive nature, rapid metastasis, and high recurrence rate [34]. Here, we showed that Tan IIA could also inhibit the migration of TNBC cells.

Then, we explore the involvement of GPER. Through extensive Western blot assay, we found that Tan IIA decreased GPER pathway-related proteins, including EGFR and ERK in a dose-dependent manner. Of note, all these effects were attenuated after the addition of GPER specific inhibitor G15, indicating a GPER-dependent mechanism. Indeed, the discovery of GPER brought a new angle to explain the effects of estrogen or estrogenic agents on cell growth and migration, especially for TNBC, in which GPER is overexpressed and associated with high recurrence rate [35]. According to a study, after 36-month follow-up, 90.5% of the TNBC patients with low GPER expression were still alive, while for GPER highly expressed patients, only 77.8% survived [36]. GPER/EGFR/ERK transduction signaling triggers c-Fos and c-Jun expressions [37–39], which could induce chromatin-activated protein 1 (AP-1) complex to promote cancer cell migration [40]. According to network pharmacology results, c-Fos and c-Jun are also potential targets of Tan IIA. Consistently, the results of the subsequent *in vitro* experiments validated that Tan IIA decreased the expression of these two proteins as well as their nucleus translocation. Therefore, GPER may be a promising drug target for TNBC.

Furthermore, the network pharmacology and in vitro experiments are consistent with that Tan IIA could influence the cell cycle, especially the G1/S transition. Cell cycle-related proteins could be potential targets for cancer therapy. For instance, CDK4/6 inhibitors, such as palbociclib and ribociclib, have been approved by FDA for the treatment of breast cancer [41, 42]. However, for TNBC, these inhibitors are ineffective, probably due to the high expression of CDK2, which leads to a greater shift of cells from S phase [43]. We chose G1/S phase-related proteins for Western blot test and showed that CDK2, CDK4, CDK6, cyclin A2, and cyclin D1 were all downregulated by Tan IIA. Cyclin A2 dominates the longest period of time in the late G1 phase and could bind to CDK2. Previous study has demonstrated that overexpression of cyclin A2 promotes TNBC proliferation, invasion, and migration while inhibiting apoptosis [44]. Cyclin D1 has been proven as an oncogenic factor since upregulation of cyclin D1 was related to poor cancer prognosis [45]. Taken together, Tan IIA showed significant inhibitory effects on a variety of cell cycle-related proteins.

## 5. Conclusions

This study suggests that Tan IIA could inhibit MDA-MB-231 cells. Importantly, these effects may be achieved through GPER/EGFR/ERK (1/2)/c-Fos and c-Jun signaling pathways. Our results provide a basis for applying Salvia miltiorrhiza in clinical practice.

## Data Availability

The data used to support the findings of this study are included within the article.
